# Exercise training reinstates cortico-cortical sensorimotor functional connectivity following striatal lesioning: development and application of a subregional-level analytic toolbox for perfusion autoradiographs of the rat brain

**DOI:** 10.3389/fphy.2014.00072

**Published:** 2014-12-03

**Authors:** Yu-Hao Peng, Ryan Heintz, Zhuo Wang, Yumei Guo, Kalisa G. Myers, Oscar U. Scremin, Jean-Michel I. Maarek, Daniel P. Holschneider

**Affiliations:** 1 Department of Biomedical Engineering, Viterbi School of Engineering, School of Medicine, University of Southern California, Los Angeles, CA, USA; 2 Department of Psychiatry and the Behavioral Sciences, Keck School of Medicine, School of Medicine, University of Southern California, Los Angeles, CA, USA; 3 Research Service, Veterans Affairs Greater Los Angeles Healthcare System, Los Angeles, CA, USA; 4 Physiology Department, David Geffen School of Medicine, University of California at Los Angeles, Los Angeles, CA, USA

**Keywords:** cerebral cortex, functional connectivity, brain mapping, exercise, motor training, Parkinson’s Disease, dopamine, software

## Abstract

Current rodent connectome projects are revealing brain structural connectivity with unprecedented resolution and completeness. How subregional structural connectivity relates to subregional functional interactions is an emerging research topic. We describe a method for standardized, mesoscopic-level data sampling from autoradiographic coronal sections of the rat brain, and for correlation-based analysis and intuitive display of cortico-cortical functional connectivity (FC) on a flattened cortical map. A graphic user interface “Cx-2D” allows for the display of significant correlations of individual regions-of-interest, as well as graph theoretical metrics across the cortex. Cx-2D was tested on an autoradiographic data set of cerebral blood flow (CBF) of rats that had undergone bilateral striatal lesions, followed by 4 weeks of aerobic exercise training or no exercise. Effects of lesioning and exercise on cortico-cortical FC were examined during a locomotor challenge in this rat model of Parkinsonism. Subregional FC analysis revealed a rich functional reorganization of the brain in response to lesioning and exercise that was not apparent in a standard analysis focused on CBF of isolated brain regions. Lesioned rats showed diminished degree centrality of lateral primary motor cortex, as well as neighboring somatosensory cortex—changes that were substantially reversed in lesioned rats following exercise training. Seed analysis revealed that exercise increased positive correlations in motor and somatosensory cortex, with little effect in non-sensorimotor regions such as visual, auditory, and piriform cortex. The current analysis revealed that exercise partially reinstated sensorimotor FC lost following dopaminergic deafferentation. Cx-2D allows for standardized data sampling from images of brain slices, as well as analysis and display of cortico-cortical FC in the rat cerebral cortex with potential applications in a variety of autoradiographic and histologic studies.

## INTRODUCTION

Rodents are primary animal models for studying the mammalian brain. Recent rodent connectome projects have begun to delineate anatomic connectivities of the rat and mouse brain with unprecedented resolution and completeness [1-3]. These connectome data clearly reveal rich and complex connectivity architectures at the subregional/mesoscopic level. How subregional structural connectivity relates to subregional functional interaction is an emerging research topic. The importance of subregional-level functional connectivity (FC) analysis is highlighted by recent reports of FC-based functional segregation within brain structures [4-6].

Correlation-based FC analysis quantifies the symmetrical statistical association between individual brain regions [7]. Two methods have been broadly used for FC analysis: inter-regional, cross-correlation analysis of time series data such as blood oxygen-level dependent signals measured with functional magnetic resonance imaging (fMRI), and inter-regional correlation analysis of cross-sectional data such as regional cerebral blood flow (rCBF) measured with positron emission tomorgraphy (PET). The latter has been applied to rodent functional brain mapping data acquired with microPET, and autoradiographic measurement of deoxyglucose uptake [8-10] and rCBF [6, 11, 12].

Study of subregional FC requires data processing of large numbers of regions-of-interest (ROIs). Animal researchers working with whole brain data sets reconstructed from tens to hundreds of serial histologic sections often face the challenge of how best to summarize data and allow for rapid exploration. We describe here a simple approach for high-density, standardized ROI definition and data extraction from autoradiographic coronal brain slices of the rat. The method allows for correlational FC and graph theoretical analysis, between-group comparison, and intuitive display of results in a flattened cortical map. Our software implementation “Cortex 2-Dimensional” (Cx-2D) was tested on a cerebral autoradiographic perfusion data set of rats that had undergone bilateral lesioning of the striatum, followed by 4 weeks of daily aerobic exercise training or no exercise. Functional brain mapping was performed in animals walking on a treadmill. Effects of lesioning and exercise on subregional FC were examined across the cortical surface.

## MATERIALS AND METHODS

We previously developed a software for the measurement, analysis and display of rCBF data obtained from autoradiographic coronal brain sections of the rat [13]. The earlier work focused on the ROI selection, measurement and statistical analysis of between-group differences in rCBF, while the current study adapted this software for the analysis of FC between brain regions.

### REGION OF INTEREST SELECTION

**FIGURE 1:**
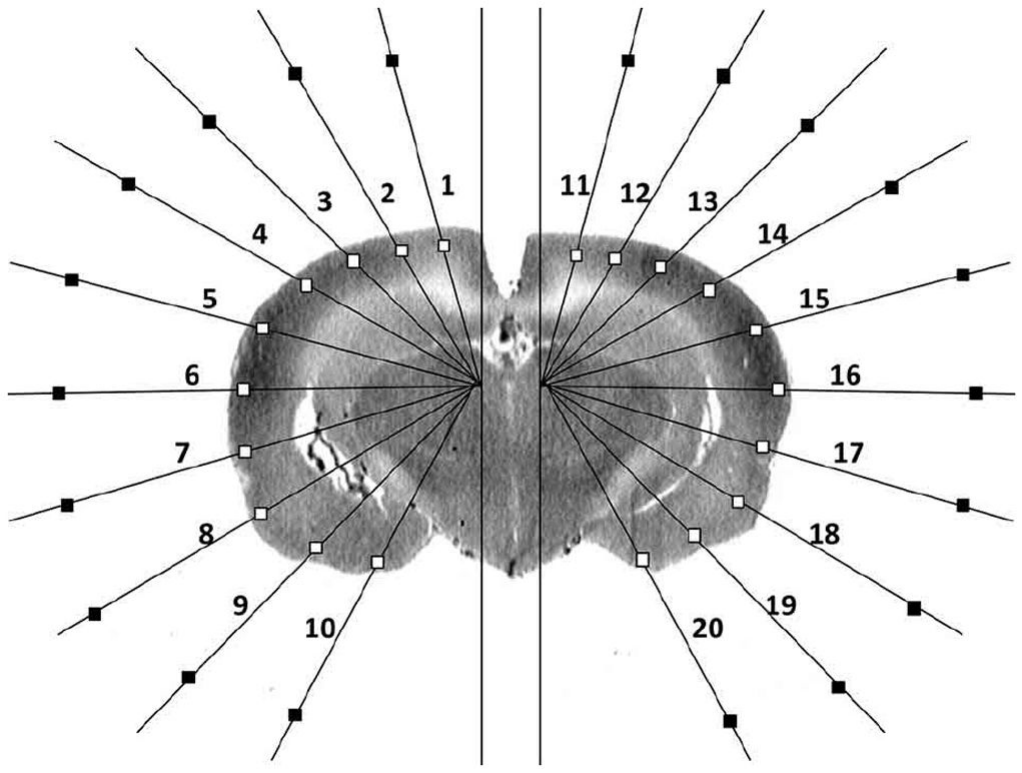
Schematic of region-of-interest and background selections in a single coronal brain autoradiographic slice as reported previously [13]

Details on the method of ROI selection can be found in our prior publication [13]. In brief, using software written in Matlab (The MathWorks, Inc., Natick, MA, USA), ROIs were sampled on 8-bit digitized brain autoradiograms using two radial, hemigrid overlays, with rays spaced in 15° intervals from the midline (Figure 1), sufficient to resolve multiple subregions within the major cortical structures. Overlay of this template on each digitized brain slice image allowed for measurement of the optical density at locations in the cortical mantle in a standardized manner across animals. Along each grid line that intersects the cortical surface, the point of intersection was identified with an algorithm that detects the edge on a binary “mask” based on a threshold gray level in the original image [13, 14]. A square ROI (default size 358 × 358 μm^2^) was placed along the ray with its center 358 μm from the intersection point. After all ROIs had been placed, the user was able to manually reposition the ROIs to avoid any artifacts that may have appeared in any given brain slice. Mean optical density was measured for each ROI in each slice (current dataset: 806 ROIs selected in 34 coronal slices in each animal, 300-μm interslice distance, beginning at 4.8 mm anterior to the bregma). For each cortical ROI, a background ROI was automatically selected in close proximity along the same radial grid line. The subtraction of the mean optical density of each ROI from that of its corresponding background ROI allowed for correction of potential inhomogeneities in the background. In the autoradiographs, a region with greater rCBF showed greater darkness but lower optical density.

Data analysis and topographic mapping of results were performed using a custom software program written in LabVIEW (National Instruments Co., Austin, TX, USA). Required user inputs included (a) the text file containing ROI optical density data, (b) a file defining for each brain a reference slice with a distinct landmark (e.g., fusion of the anterior commissures across the midline), based on which brain slices were aligned along the anterior-posterior axis across all brains, and (c) a table identifying for each bregma level the number of ROIs to be analyzed (7–10 ROIs per hemisphere). The program also used a list of brain-structure identifiers for each cell of the data matrices. These identifiers were manually derived from the overlay of the radial grids on the digitized images of the coronal brain sections from a rat brain atlas [15].

For every brain, the global mean and standard deviation (*SD*) were calculated for all ROIs in the data matrix. A Z-score transformation [16] was performed to convert optical density data into “normalized” representation of rCBF for each brain. This transformation removed variations in the global mean between brains of all groups created by global effects and systematic experimental errors. Therefore, the analysis did not account for any global differences in tracer levels that could have been present between experimental groups.

### PAIRWISE INTER-REGIONAL CORRELATION AND DEGREE CENTRALITY ANALYSIS

We applied inter-regional correlation analysis to investigate functional connectivity in the LabVIEW program. This is a well-established method, which has been applied to analyze rodent brain mapping data of multiple modalities [6, 8-12, 17-20]. Correlations were calculated across subjects within a group, and different from the within subject cross correlation analysis often used on fMRI time series data [21-24]. Pearson’s correlation coefficients between each pair of ROIs were calculated across subjects within a group for all cortical ROIs. Significant correlations (*P* < 0.05 without correction for multiple comparisons) were interpreted as functional connections. For each ROI, we then calculated degree centrality, which was defined as the number of significant correlations (positive or negative) linking it to the other ROIs. For each group, a flattened, topographic map for the cortical surface was plotted with each cell representing an ROI and the color of the cell coding the ROI’s degree. This allowed for intuitive visualization of the degree metrics for all ROIs across the cortical surface. Group differences in degree were interpreted in a qualitative manner.

### SEED ANALYSIS

To evaluate and compare the pattern of functional connectivity of individual cortical ROIs over the cortical surface, correlations of user-selected ROI seeds with all other ROIs were calculated within each group and visualized on the flattened cortical map with color-coded correlation coefficients. The threshold for significance was set at *P* < 0.05.

### TEST DATA SET

The software was tested on an autoradiographic perfusion data set that mapped brain activation during a locomotor challenge in a rat model of Parkinsonism with a 4-week aerobic exercise as intervention. A whole-brain, voxel-based analysis of changes in rCBF in this dataset has been previously reported by our group, and the reader is referred to our publication for additional details [25].

#### Animal model

The protocol was approved by the Institutional Animal Care and Use Committee (IACUC) of the University of Southern California (Protocol #11121). The animal facility at this Institution is accredited by the Association for Assessment and Accreditation of Laboratory Animal Care (AAALAC). In brief, 3-month old, male Sprague-Dawley rats were randomized into the following groups: Lesion/Exercise (*n* = 12), Lesion/No-Exercise (*n* = 10), and Sham/No-Exercise (*n* = 9). The number of animals reflects data loss due to technical issues such as cryosectioning artifact, freezer malfunction, and catheter occlusion in 3 animals. Rats received stereotaxic injection of the dopaminergic toxin 6-hydroxydopamine (10 μg 6-OHDA in 2 μL of 1% L-ascorbic acid/saline, Sigma-Aldrich Co., St. Louis, MO, USA) at four injection sites targeting the dorsal caudate putamen (striatum) bilaterally (AP: +0.6, ML: ±2.7, DV: −5.1 mm, and AP: −0.4, ML: ±3.5, DV: −5.5 mm), which resulted in ~40% of bilateral striatal volume affected, as well as a ~30 and ~38% loss in tyrosine hydroxylase optical density at the level of the striatum and substantia nigra compacta, respectively, measured by immunohistochemical staining 7 weeks after the lesion. Sham-lesioned rats received 4 injections of an equal volume of vehicle. To prevent noradrenergic effects of the toxin, rats received desipramine (25 mg/kg in 2 mL/kg bodyweight saline, i.p., Sigma-Aldrich Co.) before the start of surgery [26].

#### Exercise training

Two weeks after the lesioning, animals assigned to the exercise group were trained in a running wheel (36 rungs of 14.6 mm diameter, 4.4° angular spacing, Lafayette Instrument, Lafayette, IN, USA) for 20 min/day (4 sessions, 5 min each with 2-min inter-session intervals), 5 consecutive days/week. No-exercise animals were handled and left in a stationary running wheel for 30 min/day. Animals were trained for 4 weeks using an individually adjusted, performance-based speed adaptation paradigm as described [25]. Thereafter, rats received implantation of the right external jugular vein cannula that was externalized dorsally in the suprascapular region. Brain mapping studies occurred 4 days postoperatively.

All animals were habituated to a horizontal treadmill for 4 days prior to cerebral perfusion experiments. Each day, they were individually placed on the stationary treadmill (single lane, *L* = 50, *W* = 7, *H* = 30 cm) for 10 min followed by 3 min of walking at 8 m/min.

#### Functional brain mapping

On the day of the perfusion experiment, rats during treadmill walking at 8 m/min received a bolus intravenous administration of [^14^C]-iodoantipyrine (125 μCi/kg in 300 μL of 0.9% saline, American Radiolabeled Chemicals, St. Louis, MO, USA), followed immediately by the euthanasia agent (pentobarbital 50 mg/mL, 3 M potassium chloride). This resulted in cardiac arrest within ~10 s, a precipitous fall of arterial blood pressure, termination of brain perfusion, and death. This approach uniquely allowed a 3-dimensional (3-D) assessment of functional activation in the awake, non-restrained animal, with a temporal resolution of ~10 s and an in-plane spatial resolution of 100 μm^2^ [27, 28]. Wiping the treadmill with a 1% ammonia solution between animals minimized olfactory cues. Brains were removed, flash frozen at approximately −55°C in methylbutane on dry ice and serially sectioned for autoradiography (57 coronal 20-μm thick slices, including the cerebellum with a 300-μm interslice distance of which 34 slices were used for current analysis of the flattened cortex). Sections were exposed for 3 days at room temperature to Kodak Biomax MR film in spring-loaded x-ray cassettes along with 16 radioactive ^14^C standards (Amersham Biosciences, Piscataway, NJ). Autoradiographs were digitized on an 8-bit gray scale. CBF related tissue radioactivity was measured by the classic [^14^C]-iodoantipyrine method [29, 30]. In this method, there is a strict linear proportionality between tissue radioactivity and rCBF when the radioactivity data is captured within a brief interval (~10 s) after the radiotracer injection [31, 32].

## RESULTS

### EFFECTS OF 6-OHDA LESIONING AND AEROBIC EXERCISE ON CORTICO-CORTICAL FUNCTIONAL CONNECTIVITY NETWORK DEGREES

**FIGURE 2:**
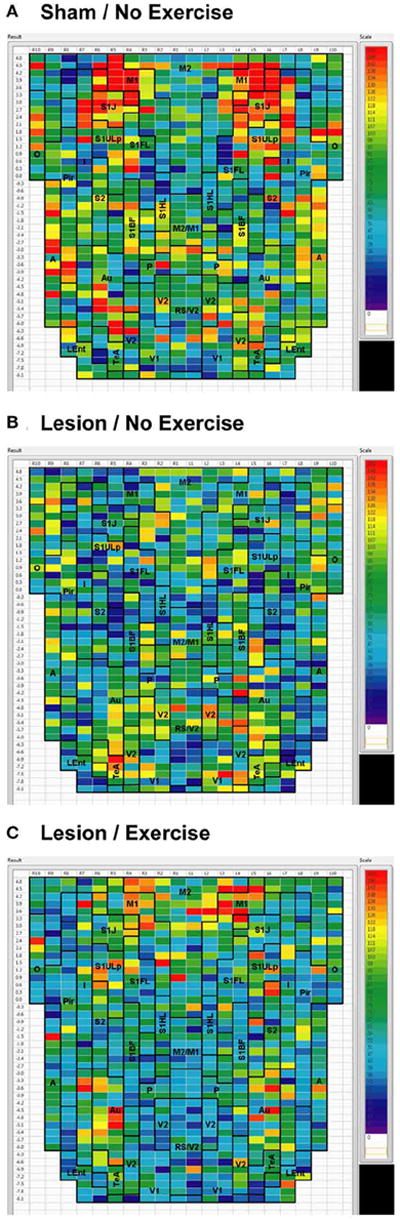
Degree of cortico-cortical functional connectivity Cortico-cortical functional connectivity degrees in animals receiving **(A)** sham treatment, **(B)** lesion without exercise, and **(C)** lesion with exercise are color-coded and shown on a flattened map of the cortical surface. The rows denote coronal sections, with ROIs represented by cells and numbered starting from the midline. Right (R) and left (L) hemispheric ROIs are shown on the *left* and *right* side of the figure, respectively. Abbreviations [15]: A, amygdala; Au, auditory; Fr3, frontal cortex area 3; I, insular; LEnt, lateral entorhinal; M1, primary motor; M2, secondary motor; O, olfactory; P, parietal; Pir, piriform; RS, retrosplenial; S1BF, primary somatosensory for the barrel fields; S1FL, forelimbs; S1HL, hindlimbs; S1J, jaw; S1ULp, upper lip region; S2, secondary somatosensory; TeA, temporal association; V1, primary visual; V2, secondary visual. Unlabel regions represent transitional areas between two regions.

**FIGURE 3:**
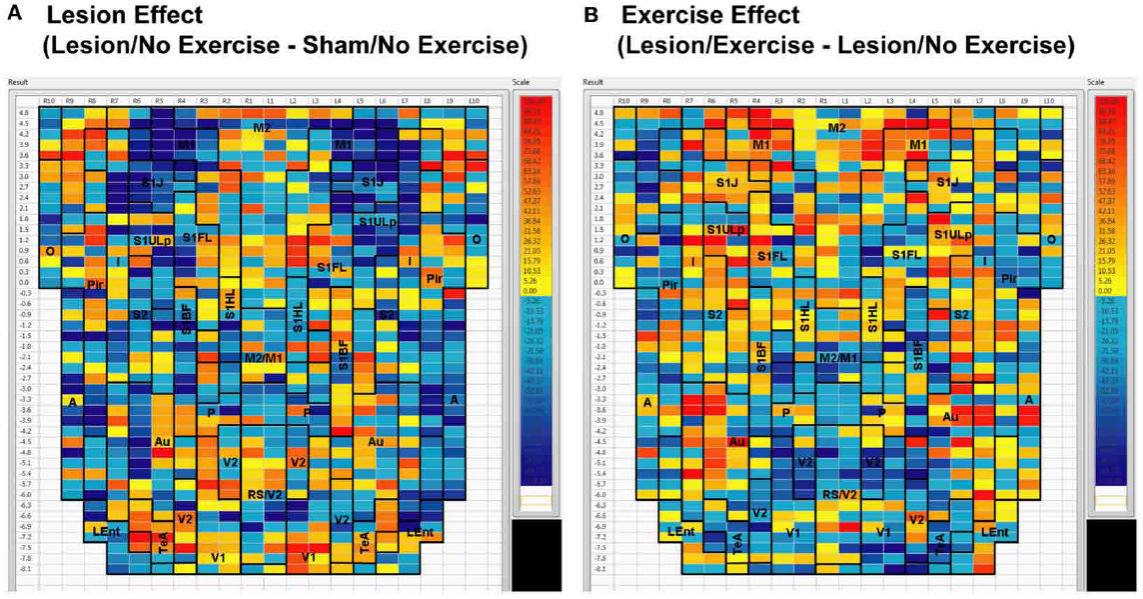
Between-group differences in functional connectivity degree Differences in cortico-cortical functional connectivity degree between **(A)** animals with bilateral striatal lesions and sham animals (Lesion/No Exercise—Sham/No Exercise) and **(B)** lesioned animals with and without exercise intervention (Lesion/Exercise—Lesion/No Exercise) are color-coded and shown on a flattened cortical map. Abbreviations are as in Figure 2.

Sham animals during walking showed the highest FC degrees in the anterior part of the primary motor cortex (M1) and in the neighboring primary somatosensory cortex, particularly in the jaw area (S1J, Figure 2A). Lesioned/no-exercise animals showed a decrease in FC degrees in these motor and somatosensory regions (Figure 2B). A map showing differences in FC degree between the sham and lesioned/no-exercise rats (Figure 3A) revealed widespread decreases in FC degree throughout M1, S1J, and the upper lip region of primary somatosensory cortex (S1ULp), as well as to a lesser extent in secondary somatosensory cortex (S2). Increases in FC degrees were observed in the anterior and ventral areas of the piriform (Pir) and olfactory/piriform transition cortex, as well as in the auditory (Au), temporal association (TeA), and posterior aspect of primary and secondary visual cortices (V1, V2, Figure 3A). Exercise training of the lesioned animals compared to lesioned/no-exercise rats resulted in an increase in FC degree in the anterior M1 and secondary motor cortex (M2). FC degree was also increased by exercise training in somatosensory areas (S1J, S1ULp, S2), while decreases were apparent in broad regions of V1, V2, and in the posterior-most aspect of M1 and M2 (Figure 3B).

### SEED CORRELATION

#### Intra-structural correlation

**FIGURE 4:**
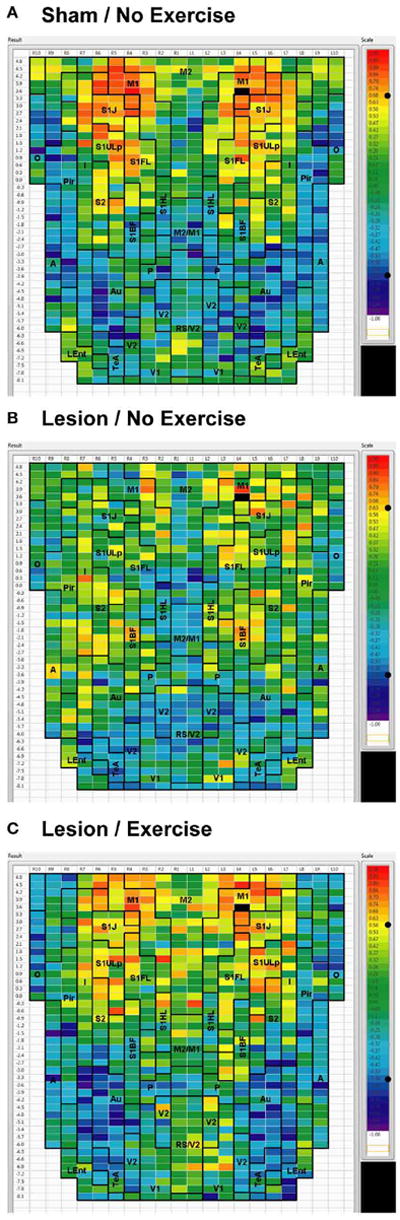
Cortical functional connectivity of the M1 seed Shown are animals receiving **(A)** sham treatment, **(B)** lesion without exercise, and **(C)** lesion with exercise. The seed is placed in the left anterior, primary motor cortical area (M1) at bregma AP + 3.6 mm (black cell on the right side of each map). Each ROI is represented by a cell with its Pearson’s correlation coefficient with the M1 seed color-coded. Positive and negative correlations are denoted by red and blue colors, respectively. The critical value of the correlation coefficient (R) for statistical significance (*P* < 0.05) is denoted by a dot (•) placed on the *R*-value color scale. Abbreviations are as in Figure 2.

**FIGURE 5:**
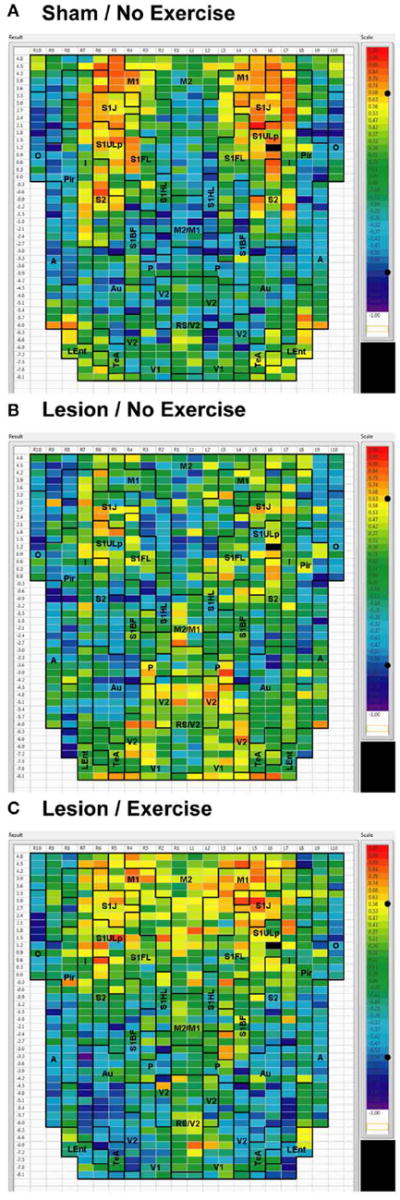
Cortical functional connectivity of the S1ULp seed Shown are animals receiving **(A)** sham treatment, **(B)** lesion without exercise, and **(C)** lesion with exercise. The seed is placed in the left anterior part of the upper lip region of the primary somatosensory cortex (S1ULp) at bregma AP +1.2 mm (black cell on the right side of each map). Each ROI is represented by a cell with its Pearson’s correlation coefficient with the S1ULp seed color-coded. Positive and negative correlations are denoted by red and blue colors, respectively. The critical value of the correlation coefficient (R) for statistical significance (*P* < 0.05) is denoted by a dot (•) placed on the *R*-value color scale. Abbreviations are as in Figure 2.

**FIGURE 6:**
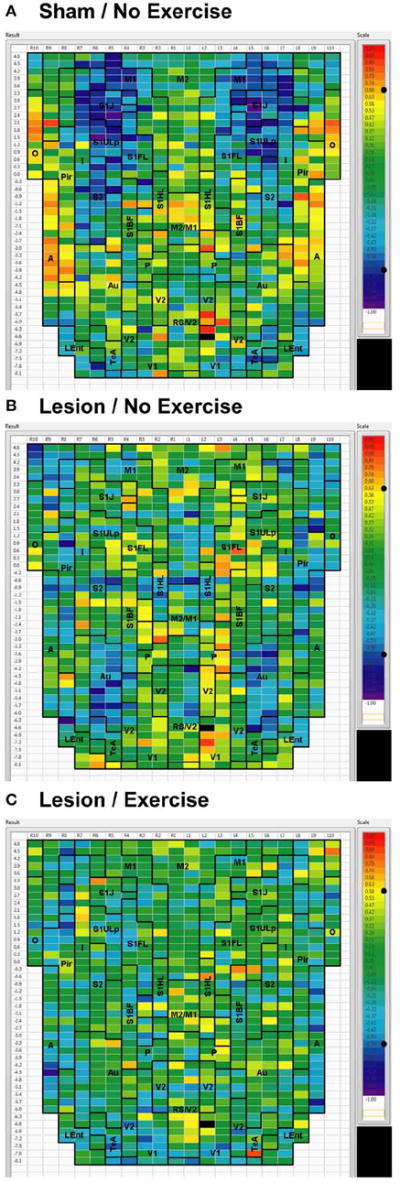
Cortical functional connectivity of the V1 seed Shown are animals receiving **(A)** sham treatment, **(B)** lesion without exercise, and **(C)** lesion with exercise. The seed is placed in the left primary visual cortex at bregma AP −6.6 mm (black cell on the right side of each map). Each ROI is represented by a cell with its Pearson’s correlation coefficient with the V1 seed color-coded. Positive and negative correlations are denoted by red blue colors, respectively. The critical value of the correlation coefficient (R) for statistical significance (*P* < 0.05) is denoted by a dot (•) placed on the *R*-value color scale. Abbreviations are as in Figure 2.

We used seed correlation analysis to explore alterations in the spatial pattern of FC of the regions showing the greatest changes in FC degree following 6-OHDA lesioning and exercise (Figures 4-6). In sham animals, a seed placed in the left M1 showed significant, bilateral, positive correlations with a large number of other M1 ROIs (Figure 4), and similar *intra-structural* (correlations between subregions within a brain structure) FC patterns were found when a seed was placed in M2, S1ULp, V1, V2, Pir or Au (Figures 5, 6, Table 1). Lesioned/no-exercise rats showed a significant loss of these intra-structural positive correlations, particularly in motor and somatosensory structures. Exercise training in lesioned animals re-established many of the intra-structural positive correlations that were lost after lesioning in areas such as M1, M2, which in fact showed greater numbers of positive intra-structural correlations than those noted in sham animals (Table 1). Similar observations were made when seeds were placed at alternate subregions within the same brain structure (data not shown).

#### Inter-structural correlation

*Inter-structural correlation* (i.e., correlations between subregions of different brain structures) also showed disruption following lesioning and recovery following exercise. For an M1 seed, lesions resulted in a decrease in the number of significant positive correlations with M2, frontal area 3 (Fr3), S1J, primary somatosensory cortex of the forelimb (S1FL), S1ULp, and S2, whereas exercise in lesioned animals increased the number of significant correlations with these structures. Importantly, the number of significant positive correlations for the M1 seed with M2, S1ULp and S2 was equal or slightly greater than those noted in the sham animals (Table 1). A similar picture was observed for the M2 seed in which lesions decreased the number of significant positive correlations with M1, Fr3, S1J, S1FL, S1ULp, and S2, and exercise increased the number of significant correlations with these structures. For the S1ULp seed, lesions decreased the number of positive correlations with M1, Fr3, S1J, S1FL, and S2, which were increased following exercise training (Figure 5, Table 1). Similar observations were made when seeds were placed at alternate ROI locations within the same brain structure (data not shown).

For the V1 seed, sham animals showed significant negative correlations to M1, S1ULp, and S1J (Figure 6A). These connections were lost in lesioned/no-exercise animals (Figure 6B) and remained absent in the lesioned/exercise animals (Figure 6C). Similar observations were made for the Au and for the Pir seeds such that lesions decreased FC with M1 and S1, which exercise did not restore (data not shown).

## DISCUSSION

While advances in the fields of human functional brain mapping have rapidly been adopted in animal imaging, several limitations remain in the application of fMRI and microPET for the functional brain mapping of rodents. Limitations center around spatial resolution, animal sedation [33-35] and animal restraint. Classic autoradiographic and histologic methods retain an important role as a means of examing whole brain functional activation with high spatial resolution in the awake, non-restrained, behaving rodent.

A dilemma faced by animal researchers working with autoradiographic or histologic datasets is how best to present whole brain data obtained from large numbers of consecutive brain sections. In the past, such data has been presented, either in table format, as individual representative slices or as summary representations on hand-drawn sketches. The current method and our prior publication described a means for the compact display of significant group differences of regional signal intensity (rCBF in the current study) and their interregional correlation. Although our method was described in relation to autoradiographic brain slice images measuring cerebral blood flow, in principle, a correlational analysis would be applicable to a wide range of modalities that use quantitative brain slice images, such as autoradiographic measurement of glucose uptake, immunohistochemical analysis of protein expression, and analysis of gene expression with *in situ* hybridization. In principle, the method for evaluating cortical FC could also be applied to the reanalysis of a vast store of data obtained from cryosections of the brain and published over the past three decades. Most of this data has not examined functional correlations between brain regions.

### METHODOLOGIC ISSUES

We described a subregional, cortico-cortical functional connectivity analysis toolbox for mapping data of the rat brain. The advantage of the current approach to FC analysis was its unbiased, semi-automated selection of large numbers of ROIs sufficient to allow detailed mapping of subregional, functional segregation. The flatmap approach to result display provided an intuitive interface to summarize FC findings across hundreds of ROIs. The representation of a brain structure by multiple subregional ROIs allowed for detection of FC differences that involve only a portion of the structure. A future improvement of this method might entail a whole-brain, voxel-based FC analysis, as has been done in human neuroimaging studies [36]. Another improvement might be to enable ROI definition and data extraction in deep midline cortical structures, including the prelimbic, infralimbic, part of cingulate, and part of retrosplenial cortices. The current framework of software allowed implementation of additional functionalities to address important functional connectivity issues. For example, flatmap display of FC could be restricted to only crosshemispheric or intrahemispheric FC, to only positive or negative correlations. More graph theoretical metrics of the cortico-cortical FC network could be calculated besides degree. The Cx-2D software could also be adapted for use in the mouse brain.

Given the large number of ROIs, we did not attempt a correction for multiple comparisons. Interpretation of our data, however, was not based on individual ROIs, but rather on patterns of change across multiple ROIs across the topographic flatmap display. Additional measures may contribute to the confidence of effects detected in a data set. Such effects may be the presence of left–right symmetry for paradigms that are intrinsically symmetrical (e.g., quadrupedal locomotion in a rat) and the correspondence of clusters of significant ROIs within the boundaries of known anatomical structures—both of which were the case for our data. These cannot be easily quantified but increase the significance of the current findings. Nevertheless, given the ongoing spirited discussion of the need for corrections for multiple comparisons in neuroimaging data, our results should be considered exploratory rather than definitive [37-39].

In our study, we applied autoradiographic perfusion mapping, with FC calculated using cross-sectional data across subjects in a group. As such, our analysis precluded evaluation of the temporal dynamics of functional brain activation. Furthermore, it is important to remember that while correlation-based analyses provide information about functional connectivity, they do not directly address causal relationships. It is possible that functional connectivity may arise in the absence of a direct structural connection, through indirect pathways or due to the influence of a common factor. Finally, although positive and negative correlations are generally interpreted as functional, neural interactions, their exact neurophysiologic substrates are not completely understood and may vary [6, 40, 41].

### EFFECTS OF DOPAMINERGIC DEAFFERENTATION AND EXERCISE TRAINING

The 6-OHDA basal ganglia injury rat model is a widely accepted model of dopaminergic deafferentation, and while not capturing all aspects of human Parkinson’s Disease (PD), parallels the human disorder remarkably well [42]. Parkinson’s patients show alterations in basal ganglia thalamocortical networks primarily due to loss of nigrostriatal dopaminergic neurons. These changes in subcortical networks lead to neuroplastic changes in motor cortex, which mediates cortical motor output. Cortical functional connectivity is impaired in PD subjects during the execution of motor tasks [43-47] and may reflect underlying abnormalities in cortical excitability [48]. The current cortico-cortical FC analysis revealed findings not initially apparent from the standard analysis of rCBF [25]. Lesions diminished much of the intra- and inter-structural FC of anterior M1 and its neighboring anterior S1 that was present in sham animals during treadmill walking. Decreases in FC were also noted in M2, however, these were more patchy. These changes were observed in the degree maps, and were confirmed using seed correlation of individual ROIs placed in M1 and S1ULp. The loss of FC across S1J, S1FL, S1ULp, and S2 was particularly apparent when only positive correlations were examined (Table 1). Lesions resulted in an increase in FC degree in dorsal areas of Au, TeA, Pir, and broadly across V1 and V2 (Figure 3B).

Exercise training in lesioned animals partially restored lesion-induced loss in FC in M1 and its neighboring somatosensory cortex, as well as in M2. This was noted both in FC degree and in seed correlation, especially with regard to positive correlations. These findings were consistent with recent reports in human subjects demonstrating increases in FC of the motor cortex following several minutes [49, 50] or 4 weeks of motor training [51]. Exercise-induced restoration of FC of the sensorimotor structures may be mediated by neuroplastic changes in motor circuits [52, 53], or normalization of corticomotor excitability [54].

## CONCLUSION

In summary, dopaminergic deafferentation of the striatum in the rat lead to diminished intra- and inter-structural positive correlations in motor and somatosensory cortex. Such abnormal sensorimotor integration has been well documented in Parkinson’s disease patients [55-57]. The altered FC in the sensorimotor structures may underlie such abnormality in our Parkinsonian rats. The disruption of cortical FC of the motor and sensory structures was partially normalized by 4 weeks of aerobic exercise training. The software Cx-2D enabled standardized, subregional ROI data extraction, functional connectivity and simple graph theoretical analysis, as well as intuitive display of FC findings. The subregional-level FC analysis and visualization in a flattened cortical map facilitated between-group comparison, as well as comparison of cortico-cortical FC with cortico-cortical anatomic connectivity as has been previously revealed by others [3]. Organizational principles learned from animal models at the macro- and mesoscopic level (brain regions/subregions and pathways) will not only inform future work at the microscopic level (single neurons and synapses), but will have translational value to advance our understanding of human brain structure and function in health and disease.

## Figures and Tables

**Table 1:** Total number of significant positive correlations of select cortical seeds with other cortical regions.

	M1 SEED			M2 SEED			S1ULp SEED		

Region	Sham	Lesion	Lesion/Ex	Sham	Lesion	Lesion/Ex	Sham	Lesion	Lesion/Ex
M1	21 (39%)	5 (9%)	20 (37%)	10 (19%)	2 (4%)	19 (35%)	16 (30%)	1 (2%)	15 (28%)
M2	4 (7%)		15 (27%)	3 (5%)	3 (5%)	29 (52%)	1 (2%)		11 (20%)
M1/M2			1 (6%)			2 (12%)		1 (6%)	
Fr3	8 (100%)		6 (75%)	4 (50%)	1 (12%)	6 (75%)	6 (75%)	1 (12%)	5 (62%)
S1J	15 (75%)	1 (5%)	6 (30%)	10 (50%)	1 (5%)	4 (20%)	12 (60%)	3 (15%)	12 (60%)
S1FL	11 (29%)	1 (3%)	8 (21%)	3 (8%)		9 (24%)	8 (21%)	1 (3%)	6 (16%)
S1ULp	10 (33%)	1 (3%)	12 (40%)	4 (13%)	1 (3%)	9 (30%)	11 (37%)	2 (7%)	3 (10%)
S2	1 (5%)		5 (23%)	6 (27%)		7 (32%)	7 (32%)	2 (9%)	3 (14%)
V1		1 (3%)	2 (5%)			1 (3%)		3 (8%)	1 (3%)
V2			2 (7%)			2 (7%)		6 (20%)	1 (3%)
Au									
Pir				6 (10%)					1 (2%)

	V1 SEED			Pir SEED			Au SEED		

Region	Sham	Lesion	Lesion/Ex	Sham	Lesion	Lesion/Ex	Sham	Lesion	Lesion/Ex

M1		1 (2%)	2 (4%)						
M2		1 (2%)					2 (4%)		
M1/M2	3 (19%)		2 (12%)	1 (6%)			1 (6%)		
Fr3			1 (12%)						
S1J		1 (5%)	1 (5%)						
S1FL	1 (3%)	4 (11%)							
S1ULp		1 (3%)	1 (3%)						
S2					4 (18%)	2 (9%)			1 (5%)
V1	4 (11%)	5 (13%)		4 (11%)			9 (24%)	3 (8%)	1 (3%)
V2	3 (10%)			1 (3%)			7 (23%)	4 (13%)	3 (10%)
Au	1 (2%)		1 (2%)		1 (2%)	2 (5%)	13 (30%)	20 (45%)	21 (48%)
Pir	11 (18%)		1 (2%)	20(32%)	6 (10%)	10 (16%)	6 (10%)	2 (3%)	3 (5%)

Left hemispheric seeds were chosen in primary motor cortex (AP +3.6 mm), secondary motor cortex (AP +4.5 mm), primary somatosensory cortex (upper lip region, AP +1.2 mm), primary visual cortex (AP −6.6 mm), piriform cortex (AP −1.8 mm) and auditory cortex (AP −4.8 mm). Shown are the number of positive correlations and their representation as a percentage of the total number of brain regions with the same region identifier (% rounded to nearest integer), with empty cells indicating absence of significant correlation. Gray shaded cells show an increase in positive correlations of 11–20%, whereas black shaded regions show an increase greater than 20%. Abbreviations are as in Figure 2.
